# Beyond Amyloid: Targeting Co-Aggregating Proteins and Targeted Degradation Strategies in Alzheimer’s Disease

**DOI:** 10.3390/biomedicines14010216

**Published:** 2026-01-19

**Authors:** Martina Monaco, Alessandra Pinto, Massimo Grilli

**Affiliations:** 1 Pharmacology and Toxicology Unit, Department of Pharmacy, School of Medical and Pharmaceutical Sciences, University of Genoa, Viale Cembrano 4, 16148 Genoa, Italy; martina.monaco@edu.unige.it (M.M.); alessandra.pinto@edu.unige.it (A.P.); 2 IRCCS Ospedale Policlinico San Martino, 16132 Genoa, Italy

**Keywords:** Alzheimer’s disease, amyloid-β, protein co-aggregation, targeted protein degradation, PROTACs, LYTACs, molecular glues, midkine, pleiotrophin, clusterin

## Abstract

Alzheimer’s disease (AD) involves a constellation of molecular processes that extend well beyond amyloid-β (Aβ) accumulation. Recent anti-amyloid antibodies provide limited clinical benefits, highlighting the need for additional strategies due to their modest efficacy and safety concerns. Increasing proteomic evidence reveals that proteins such as midkine (MDK), pleiotrophin (PTN) and clusterin (CLU) accumulate within amyloid plaques and may shape disease progression, although their precise contributions—protective, pathogenic, or both—remain unknown. In this Perspective, we examine how emerging targeted protein degradation (TPD) technologies, including Proteolysis-Targeting Chimeras (PROTACs), Lysosome-Targeting Chimeras (LYTACs) and molecular glues (MGs), could provide a means to selectively eliminate these co-aggregating proteins. We also discuss advances in degrader design, artificial intelligence (AI)-assisted screening, and strategies aimed at enhancing Central Nervous System (CNS) delivery. We finally outline how integrating TPD modalities with antibody-based and multi-target therapeutic approaches may promote more effective, systems-level interventions for AD.

## 1. Introduction

Alzheimer’s disease (AD) remains one of the major challenges in both neurodegenerative research and clinical practice. Despite decades of effort, therapeutic advances remain limited. Monoclonal antibodies such as Lecanemab and Donanemab reduce amyloid burden and modestly slow cognitive decline, offering cautious optimism [1,2]. However, the overall clinical impact remains limited rather than transformative. High treatment costs, the need for repeated infusions, and the risk of amyloid-related imaging abnormalities further restrict their widespread use [3,4]. Crucially, modest efficacy despite substantial clearance suggests that amyloid burden alone does not explain cognitive decline [5,6,7]. One plausible explanation is that aggregation dynamics and conformational states, rather than plaque burden alone, shape the persistence of neurotoxic amyloid-β (Aβ) species and downstream dysfunction.

This view has redirected attention towards alternative molecular players that may modulate the trajectory of the disease more effectively. Among these, co-aggregating proteins, molecules that interact with Aβ and influence its aggregation, are emerging as promising candidates. They fill a conceptual gap between amyloid deposition and downstream targets (tau spread, neuroinflammation) by tuning assembly pathways, plaque composition, and local cellular responses. Recent studies indicate that midkine (MDK) and pleiotrophin (PTN) modulate Aβ aggregation dynamics, with context-dependent effects ranging from attenuation to facilitation [8,9,10]. These proteins have been linked to neuroinflammation, oxidative stress, and synaptic dysfunction [9]. Despite this pathogenic relevance, strategies to selectively neutralize or eliminate these proteins remain underdeveloped. Notably, therapeutic approaches have evolved to encompass seemingly contradictory strategies—from promoting aggregation clearance to preventing initial assembly—reflecting the multifaceted nature of amyloid pathology [11,12].

We propose that focusing on the co-aggregation of proteins could enhance current therapeutic strategies for diseases like AD. By moving beyond therapies that only target amyloid formations, we may improve treatment outcomes. We discuss the rationale for this approach and how targeted protein degradation (TPD) technologies can selectively eliminate these proteins. Additionally, we explore integrating these strategies with existing therapies for more effective multi-target interventions.

## 2. State of the Art: Protein Co-Aggregation in AD

The aggregation of Aβ peptides has long been framed as a central event in AD pathogenesis [13,14]. Nonetheless, the amyloid hypothesis has been repeatedly challenged [15,16], and the modest clinical impact of anti-amyloid therapies underscores the need for a broader, more integrative view. On the other hand, it would be unwise to completely ignore the potential for amyloid dysregulation. It is imperative to investigate the dynamics of amyloid in relation to new and emerging partners. Indeed, recent evidence suggests that Aβ does not act in isolation, but rather its assembly is modulated by a network of co-aggregating proteins that influence nucleation, fibril growth, and plaque maturation. These molecules, often called “amyloid chaperones” or part of an “amyloid responsome,” influence the aggregation process and change the structural and functional characteristics of Aβ assemblies. This, in turn, has significant implications for neurotoxicity. The “amyloid responsome” refers to a collection of plaque-associated partners that interact with and affect aggregation as well as the handling of the microenvironment. This concept builds upon previous lists of co-localized amyloid-associated proteins.

Over the past three years, proteomic and interactomic studies have identified several key players in this process. MDK and PTN, two heparin-binding growth factors, have emerged as potent modulators of Aβ aggregation [10,17]. Both proteins bind Aβ oligomers with high affinity, promoting fibrillogenesis and stabilizing toxic conformations. Recent data now indicate, however, that MDK, despite its robust enrichment within amyloid plaques and its tight correlation with Aβ levels, can act as a context-dependent modulator of aggregation. In biophysical and in vivo models, MDK attenuates Aβ fibril assembly and reduces plaque formation, whereas genetic loss of MDK exacerbates amyloid deposition and microglial activation. Research suggests that MDK acts as a binding-dependent “buffer” for Aβ assembly. Its effects depend on factors like concentration, disease stage, and the environment of the plaques. This situation may seem contradictory, but it shows a broader trend among co-aggregating proteins. Initially, these proteins may help respond to stress, but as disease progresses, they can contribute to worsening pathology when plaques are present for a long time. This dual behavior highlights the changing and complex nature of proteins in the plaque environment. Understanding these mechanisms is important for treatment. Therapies should aim to protect helpful soluble proteins while also targeting harmful species related to the plaques.

Clusterin (CLU; apolipoprotein J), a well-known extracellular chaperone, further illustrates this complexity. Implicated in both plaque formation and clearance, CLU maps to the highly conserved M42 matrisome module identified through integrative proteomics across human AD brain tissue and Aβ-depositing mouse models [17]. Historically considered protective, CLU is now understood to display context-dependent roles [17]; under conditions of chronic inflammation, for example, it may facilitate the deposition of insoluble aggregates rather than their removal. Recent research has shown that Apolipoprotein E (APOE) can form fibrillar aggregates within microglia, which may trigger β-amyloidosis. This finding indicates that proteins that co-aggregate with APOE might not only influence existing amyloid pathology but could also play a role in initiating the pathological cascade. This perspective adds to our understanding of genetic risk models centered around complement isoforms by suggesting that, in addition to their isoform-dependent functions, APOE may also be involved in an aggregation-prone mechanism that contributes to the onset of pathology. Beyond APOE, the extracellular matrix (ECM) protein vitronectin was identified in senile plaques several years ago [18,19].

Consistent with their proposed role as stress responders, these proteins may become pathogenic primarily when chronically dysregulated, thereby coupling altered co-aggregation to sustain inflammatory and synaptic network disruption. Collectively, these interactions affect multiple pathways beyond amyloid pathology. Co-aggregating proteins have been linked to enhanced neuroinflammatory signaling, oxidative stress, and synaptic dysfunction. MDK, for example, activates microglial receptors and promotes cytokine release, creating a pro-inflammatory environment that worsens neuronal injury [20]. CLU has been associated with complement activation and altered lipid metabolism, which further contributes to network-level disruptions in neuronal connectivity [21]. Despite these insights, therapeutic strategies that directly target co-aggregating proteins remain scarce, creating an opportunity to modulate aggregation and downstream toxicity more effectively than amyloid clearance alone.

## 3. Emerging Technologies for Selective Degradation

TPD offers a fundamentally different therapeutic strategy for challenging proteins. Rather than inhibiting protein function, TPD technologies co-opt endogenous clearance systems to remove the protein entirely [22]. Among the modalities developed to date, two have received the most attention: PROTACs (Proteolysis Targeting Chimeras), which engage the ubiquitin–proteasome system, and LYTACs (Lysosome Targeting Chimeras), which direct extracellular or membrane-associated proteins toward lysosomal degradation [23]. LYTACs function as a bridge, connecting extracellular or membrane-associated proteins to the endo-lysosomal system. They achieve this by recruiting lysosome-targeting receptors that facilitate the internalization and delivery of substrates to the lysosome, which are typically inaccessible to the proteasome. Importantly, the effectiveness of LYTAC-mediated degradation has been demonstrated for extracellular and cell-surface targets only in laboratory settings. However, applying this approach to CNS diseases is still an area of ongoing research [24]. In addition, lysosomal flux and degradative capacity may be impaired in aging and AD, raising the possibility of saturation effects that could constrain lysosome-directed modalities in diseased brains. Complementary approaches, such as molecular glues (MGs), which are increasingly designed with artificial intelligence (AI) support, further expand the toolkit [25]. In the context of neurodegenerative diseases such as AD, these technologies offer the theoretical advantage of removing “undruggable” proteins, including co-aggregating chaperones, extracellular factors, or aggregation-prone proteins for which small-molecule inhibitors are ineffective [22,25].

PROTACs are bifunctional small molecules that bridge a protein of interest (POI) and an E3 ubiquitin ligase. A linker facilitates the connection between the two modules. This process results in the ubiquitination of the POI and subsequent degradation via the proteasome. Degradation being a catalytic process, PROTACs frequently necessitate sub-stoichiometric dosing [26,27].

LYTACs, by contrast, exploit lysosomal pathways to eliminate extracellular or membrane proteins that are inaccessible to the proteasome. By linking a POI-binding moiety to a ligand for lysosome-shuttling receptors (such as the cation-independent mannose-6-phosphate receptor), LYTACs redirect the target into the endosomal-lysosomal trafficking route [28]. This mechanism dramatically broadens the repertoire of degradable substrates. Beyond soluble intracellular proteins, LYTACs can in principle remove extracellular chaperones, plaque-associated cofactors or misfolded proteins trapped within the interstitial environment, providing a conceptual bridge to modulating the plaque microenvironment itself [26]. Modifying the amyloid plaque microenvironment using LYTACs is a plausible hypothesis, though it remains unvalidated and carries significant translational risks. Major uncertainties include the accessibility of targets within the plaques, the availability of receptors in CNS cell types, and the challenges related to delivering heterobifunctional constructs [29]. Future validation could leverage plaque proteomics (pre/post-treatment) to quantify depletion of co-aggregators within deposits. Complementary readouts could include CSF/plasma biomarkers and in vivo imaging of plaque burden and neuroinflammation to link target engagement to biological effects.

MGs are a third, smaller and structurally simpler class of degraders. Rather than bridging two proteins, they stabilize novel interactions between the POI and an E3 ligase, converting a weak or transient association into a productive degradation signal [25,26,30]. In comparison to PROTACs, their smaller size and simpler structure frequently result in more favorable pharmacokinetics and potentially enhanced BBB penetrance, which is a critical advantage for neurodegenerative targets [31].

### Advancements in Degrader Design and Screening

Recent advances in AI and machine learning (ML) have rapidly transformed the design of targeted protein degraders. Beyond their structural advantages, the design of MGs, and indeed all TPD modalities, has been revolutionized by the advent of sophisticated computational platforms. This technological advancement has concomitantly rendered possible the prediction of optimal linker architectures and the evaluation of E3 ligase compatibility and target engagement. For instance, DeepPROTAC, a deep-learning framework trained to predict PROTAC-induced ternary complexes, has demonstrated substantial improvements in identifying productive POI-E3 geometries and in prioritizing linker topologies [32]. Similarly, AlphaFold3 (AF3)-based pipelines, incorporating AF3-multimer and hybrid molecular-dynamics workflows, have been successfully utilized to predict POI-PROTAC-E3 assemblies with a level of experimental accuracy, offering powerful tools for degrader pre-screening and hit triage [33,34]. These AI-driven strategies accelerate early-stage discovery. Nonetheless, in silico prioritization remains hypothesis-generating and requires extensive experimental validation, particularly for CNS-relevant targets and exposure constraints. Furthermore, ML models are increasingly being used to identify previously unrecognized E3 ligase recruiters, expand degrader chemical space and engineer compounds with more favorable pharmacokinetic and pharmacodynamic profiles. As these technologies continue to mature, it is expected that they will fundamentally reshape the rational development of next-generation degradation modalities [35,36,37]. Importantly, these computational tools could also accelerate the design and triage of degradation architectures for extracellular or plaque-associated targets by prioritizing binding modes and geometries compatible with selective engagement in the plaque microenvironment.

Despite the conceptual appeal of TPD for neurodegeneration, translating these technologies to the Central Nervous System (CNS) disorders faces formidable obstacles. A major limitation is the difficulty many degraders face in crossing the BBB, as their size and physicochemical properties often result in inadequate CNS penetration [31]. This obstacle poses a significant bottleneck, particularly for heterobifunctional degraders such as PROTACs, which frequently exceed the molecular weight range compatible with efficient BBB transport. MGs may be more appealing for CNS applications due to their simpler, monovalent structure, which enhances brain penetration compared to larger degraders. However, effective delivery also depends on cell-type-specific uptake and target engagement within the CNS, as challenges arise when addressing different cells like neurons and glial cells and specific areas such as perivascular niches.

Another important consideration is specificity: recruiting E3 ligases to selectively degrade a protein of interest (POI) must be executed carefully to prevent off-target ubiquitination of non-disease proteins, as this could result in toxicity. MGs, with their more simplified architecture, serve to reduce some complexity but may also induce unwanted protein–protein interaction with undesired targets [30].

To date, most successful TPD applications—particularly PROTACs and MGs—have emerged from oncology, where intracellular targets and peripheral bioavailability are more accessible. By contrast, CNS applications remain at an earlier developmental stage. Kuemper and colleagues (2024) emphasized that, although degraders theoretically hold great promise for neurodegeneration, major translational barriers persist, with drug delivery and BBB permeability at the forefront [22]. A notable exception is ARV-102, the first brain-penetrant PROTAC to enter clinical development for neurodegeneration. Designed to degrade LRRK2 in Parkinson’s disease, ARV-102 has demonstrated that it is indeed possible to achieve CNS-directed degradation in humans, providing an important proof of concept for the field [31]. Current TPD efforts in neurodegeneration still focus primarily on intracellular targets such as tau or α-synuclein [27,28,31]. While these advances are encouraging, a major gap remains: there is still no experimental evidence that extracellular AD-relevant co-aggregating proteins can be selectively degraded using TPD platforms. Achieving this will require degraders that are not only highly specific but also capable of navigating the extracellular microenvironment and, ideally, exhibiting efficient brain penetration. Developing next-generation degraders that meet these requirements represents a critical unmet need and a major frontier for therapeutic innovation in AD.

## 4. Integration with Other Therapeutic Strategies

The complexity of AD—now widely recognized as a pathologically heterogeneous condition involving systemic dysfunction and the accumulation of multiple misfolded proteins—calls for therapeutic strategies that move beyond single-target interventions. TPD modalities offer a catalytic mechanism to remove neurotoxic proteins, and their integration with existing treatment approaches may yield synergistic effects by addressing several facets of the disease simultaneously. In such combinations, pharmacodynamic interactions and sequencing (e.g., debulking extracellular aggregates versus degrading intracellular drivers) may influence both efficacy and safety and will require empirical optimization.

### 4.1. Combination with Anti-Amyloid and Anti-Tau Antibodies

Immunotherapy approaches utilize antibodies to promote clearance of extracellular [38,39] and intracellular aggregates [40]. Recent monoclonal antibodies, like Aducanumab and Lecanemab, specifically target fibrillar or prefibrillar species [41,42,43]. While these agents primarily act by promoting aggregate removal or preventing further assembly, TPD technologies excel at degrading intracellular targets such as tau proteins for which traditional inhibitors are inadequate.

One challenge in deploying PROTACs for CNS diseases is their large molecular weight, which compromises BBB penetration [44]. Hybrid strategies have been introduced to overcome this limitation. PROTAC-Antibody Conjugates (PACs), for instance, couple degraders to monoclonal antibodies that exploit receptor-mediated transcytosis (RMT) to access the brain. PACs directed toward the transferrin receptor exemplify how tau-targeting degraders might be delivered effectively across the BBB, linking a powerful degradation mechanism to well-established biologic delivery platforms [44,45]. Mechanistically, the antibody moiety acts as a BBB ‘shuttle’ by binding an RMT receptor (e.g., TfR), enabling endothelial uptake and transcytosis. Linker chemistry (e.g., cleavable designs) is then intended to favor payload release and downstream intracellular engagement of the degradation machinery in the brain. However, PAC translation may be constrained by immunogenicity risk and manufacturing/formulation complexity typical of antibody–payload conjugates, which may impact scalability and regulatory development. LYTACs, inherently capable of directing extracellular and membrane-bound proteins to lysosomes, provide a complementary strategy that may enhance clearance pathways already engaged by therapeutic antibodies, especially those relying on lysosomal shuttling.

### 4.2. Possible Synergy with Immunomodulation and Metabolic Therapies

AD pathogenesis is intrinsically linked to chronic neuroinflammation and oxidative stress [46,47,48]. These findings suggest potential synergy between TPD and therapies targeting these systemic stressors. Conceptually, some combinations may primarily deliver symptomatic or resilience benefits (e.g., synaptic support), whereas degradation of aggregation drivers aims at disease modification; distinguishing these endpoints will be important in trial design. Antiox-PROTACs can be viewed as a multi-target delivery-aware paradigm that couples neurotoxic protein degradation with mitigation of chronic oxidative stress, a systemic driver of AD pathology.

A notable hybrid approach is the Antiox-PROTAC paradigm, which structurally integrates a neuroprotective natural antioxidant compound (e.g., a flavonoid) directly into the PROTAC molecule [49]. This single molecule simultaneously employs the TPD mechanism to degrade neurotoxic proteins (such as tau) and utilizes the antioxidant moiety to mitigate the underlying chronic oxidative stress and neuroinflammation that accelerate disease progression. Furthermore, combining TPD with immunomodulation, strategies aimed at restoring neuronal homeostasis and synaptic plasticity, often mediated by microglia and astrocytes, is appealing [50]. Just as monoclonal antibodies modulate immune responses to reduce neuroinflammation, combining TPD for aggregated proteins with agents that enhance neurotrophic factors (like BDNF and NGF) represents a comprehensive strategy to enhance neuronal survival and neuro-regeneration.

### 4.3. Multi-Target Approaches

The shift away from monotherapy stems from the realization that amyloid and tau pathology rarely exist in isolation [51]. The brain often exhibits co-pathologies, including the accumulation of TDP-43, which complicates the condition and drives the necessity for complex therapeutic intervention [52].

Multi-target-directed ligands (MTDLs) and multi-target drugs are strategic avenues to address these complex, interconnected pathological processes, which include protein aggregation, oxidative stress, neuroinflammation, and synaptic dysfunction [6]. The rationale is that simultaneously modulating multiple biological targets through a single agent or combination therapy achieves potentially greater efficacy than pursuing a single target alone. TPD modalities are inherently flexible within this framework: the PROTAC platform itself is utilized in hybrid models (like Antiox-PROTACs) that address both the molecular aggregates (tau) and systemic drivers (oxidative stress) in a single compound, providing a comprehensive framework for disease modification. A practical consideration is whether current regulatory pathways are fully equipped to evaluate increasingly complex multi-target designs (and combinations) requiring integrated evidence on component contributions, safety, and biomarker-linked mechanisms.

## 5. Future Perspectives

The field of TPD remains in its early stages for neurodegenerative disorders. While degraders have advanced rapidly in oncology, several challenges still constrain their application in the CNS, underscoring the need for methodological innovation, careful translational planning and a decisive shift toward precision medicine. Progress in these areas will be essential to transform TPD from a conceptual framework into a clinically actionable strategy for AD. In the near term, CNS delivery and exposure represent the primary bottleneck, followed by selectivity and safety; biomarker frameworks and trial designs are essential enablers for translation, whereas fully personalized, multi-omic matching remains a longer-term aspiration.

### 5.1. Research Directions: High-Capacity Screening, 3D Cell Models, and AI

Future progress in CNS-directed TPD development will depend heavily on technologies that accelerate discovery, validation and mechanistic understanding. High-throughput screening and structure-based drug design remain central tools for efficiently identifying chemical matter with degrader potential.

AI and ML are becoming particularly influential. These approaches can integrate structural and biochemical data to predict target-ligase compatibility, model degrader-induced ternary complexes and refine candidate molecules with improved pharmacokinetic and pharmacodynamic properties. AI-driven tools have demonstrated exceptional capacity for navigating complex molecular datasets, thereby overcoming several historical bottlenecks in drug development. These tools can effectively aid in prioritizing E3 ligases by ranking productive POI–E3 geometries and optimizing degrader designs for selectivity-favoring ternary complexes.

Equally important is the reliance on experimental systems that more accurately recapitulate human CNS pathology. Patient-derived induced pluripotent stem cells, organoid cultures and engineered cell lines represent powerful platforms for evaluating the interactions between degraders, E3 ligases and pathogenic proteins in biologically relevant environments. These models also enable targeted screening for MG activity and help to validate candidate ligases suitable for CNS-restricted degradation strategies. Notably, they can be adapted to plaque-like microenvironments (e.g., Aβ seeding/3D matrices) to interrogate how co-aggregating proteins are recruited and whether degradation strategies can selectively modulate these plaque-associated pools.

### 5.2. Regulatory and Clinical Challenges: Safety, Adaptive Trials, and Biomarkers

The transition from preclinical proof of concept to clinical efficacy requires navigating several translational hurdles. BBB permeability remains a dominant challenge, especially for large heterobifunctional degraders, and strategies to improve CNS delivery must remain a priority.

Safety and selectivity: enhancing target selectivity is paramount to maximizing the therapeutic index. Currently, TPD research relies heavily on promiscuous E3 ligases like VHL and CRBN, increasing the risk of off-target effects. Because these ligases are broadly expressed, CNS applications may be particularly vulnerable to systemic liabilities unless tissue-biased ligase engagement can be achieved. The focus must shift toward validating and exploiting novel, CNS-enriched E3 ligases (such as RNF182, which is upregulated in AD patients) to improve tissue specificity and reduce systemic toxicity [7,53,54]. There is also a critical need for PROTACs and other degraders capable of achieving differential selectivity, degrading the pathological, aggregated forms of proteins (like hyperphosphorylated tau or TDP-43 aggregates) while sparing the beneficial physiological forms [51]. In early-phase trials, this could be operationalized by demonstrating target engagement with species-resolved biomarker readouts (e.g., aggregate-enriched fractions or PTM-specific assays) alongside exposure–response relationships and safety monitoring.

Clinical strategy and biomarkers: successful clinical implementation demands improved clinical trial design. Crucially, novel approaches require the identification and validation of new biomarkers to track disease progression and therapeutic response. Advanced multi-omics capabilities (genomics, transcriptomics, and proteomics, including quantitative Mass Spectrometry Proteomics) are essential tools for identifying and validating these biomarkers, aiding in patient stratification and response prediction.

### 5.3. Long-Term Vision: Personalized Therapies

AD is increasingly recognized as a biologically heterogeneous disorder shaped by genetic background, molecular signatures and the presence of multiple co-aggregating proteins, including TDP-43 and α-synuclein [51]. This recognition has accelerated the movement toward personalized therapeutic strategies that account for individual variations in disease drivers.

Large-scale deep proteomic studies have been instrumental in revealing distinct molecular subtypes of AD, delineating patterns that extend well beyond the classical amyloid–tau framework. These datasets capture differences in immune activation, extracellular matrix remodeling, synaptic vulnerability and metabolic stress, offering a high-resolution view of disease heterogeneity. When integrated with predictive modelling, such multi-omic information could guide the design of customized multi-target interventions, matching individual patients with degrader strategies that address their specific constellation of pathological proteins and systemic dysfunctions. Ultimately, the promise of precision TPD lies in its flexibility: the ability to tailor degradation mechanisms to each patient’s molecular profile may enable disease-modifying therapies that address the full pathological complexity of NDDs, rather than isolated components of the disease cascade. Finally, highly personalized approaches raise ethical, economic and accessibility considerations—particularly regarding cost, infrastructure requirements and equitable patient access—that should be anticipated as the field matures.

## 6. Conclusions

**Figure 1:**
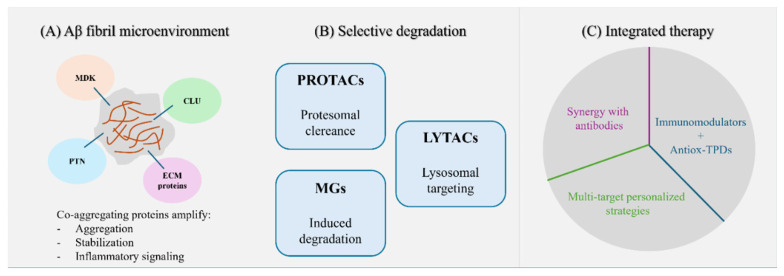
(**A**) Aβ fibril microenvironment. Co-aggregating proteins (MDK, PTN, CLU, ECM components) enhance Aβ aggregation, fibril stabilization and inflammatory signaling. (**B**) Selective degradation strategies. PROTACs promote proteasomal clearance; LYTACs enable lysosomal targeting; MGs induce TPD. (**C**) Integrated therapeutic approaches. Combined strategies include antibody synergy, immunomodulators with antioxidants/TPDs and personalized multi-target interventions.

Targeting co-aggregating proteins and harnessing TPD technologies represents a conceptual shift beyond traditional amyloid-centric strategies. Integrating PROTACs, LYTACs, MGs, and AI-driven design with immunomodulatory, metabolic, and multi-target therapeutic strategies allows for a more comprehensive approach to addressing the multifactorial nature of Alzheimer’s disease (Figure 1).

Progress, however, will depend on resolving several outstanding challenges. At the mechanistic level, clarifying the context-dependent functions of MDK and PTN in amyloid dysregulation remains essential. Experimentally demonstrating that extracellular co-aggregating proteins can be selectively degraded is another major prerequisite. Equally critical is the development of reliable pharmacodynamic biomarkers to track target engagement and the creation of brain-penetrant degraders with acceptable safety profiles. Accordingly, we call for the field to prioritize rigorous proof-of-concept studies for extracellular/plaque-associated degraders in AD-relevant models, combining exposure confirmation with plaque proteomics and biomarker-linked target engagement readouts.

Only through systematic resolution of these challenges will TPD-based approaches mature into clinically viable strategies. If successfully realized, such advances have the potential to reshape therapeutic paradigms for AD, offering interventions that act not on a single pathway but on the interconnected network of molecular events that drive neurodegeneration.

## Data Availability

No new data were created or analyzed in this study.

## Abbreviations

The following abbreviations are used in this manuscript:

AD	Alzheimer’s disease
Aβ	Amyloid-β
MDK	Midkine
PTN	Pleiotrophin
CLU	Clusterin
TPD	Targeted protein degradation
PROTACs	Proteolysis-Targeting Chimeras
LYTACs	Lysosome-Targeting Chimeras
MGs	Molecular glues
AI	Artificial Intelligence
CNS	Central Nervous System
APOE	Apolipoprotein E
ECM	Extracellular matrix
BBB	Blood–brain barrier
POI	Protein of interest
ML	Machine learning
MTDLs	Multi-target-directed ligands
PACs	PROTAC-Antibody Conjugates
RMT	Receptor-mediated transcytosis
AF3	AlphaFold3
